# Genomic insights into the taxonomic status of the *Bacillus cereus* group

**DOI:** 10.1038/srep14082

**Published:** 2015-09-16

**Authors:** Yang Liu, Qiliang Lai, Markus Göker, Jan P. Meier-Kolthoff, Meng Wang, Yamin Sun, Lei Wang, Zongze Shao

**Affiliations:** 1 State Key Laboratory Breeding Base of Marine Genetic Resources; Key Laboratory of Marine Genetic Resources, Third Institute of Oceanography, SOA; South China Sea Bio-Resource Exploitation and Utilization Collaborative Innovation Centre; Fujian Collaborative Innovation Center for Exploitation and Utilization of Marine Biological Resources; Key Laboratory of Marine Genetic Resources of Fujian Province, Xiamen 361005, China; 2 Leibniz Institute DSMZ—German Collection of Microorganisms and Cell Cultures GmbH, Inhoffenstraβe 7B, 38124, Braunschweig, Germany; 3 TEDA School of Biological Sciences and Biotechnology Nankai University, Tianjin, China

## Abstract

The identification and phylogenetic relationships of bacteria within the *Bacillus cereus* group are controversial. This study aimed at determining the taxonomic affiliations of these strains using the whole-genome sequence-based Genome BLAST Distance Phylogeny (GBDP) approach. The GBDP analysis clearly separated 224 strains into 30 clusters, representing eleven known, partially merged species and accordingly 19–20 putative novel species. Additionally, 16S rRNA gene analysis, a novel variant of multi-locus sequence analysis (nMLSA) and screening of virulence genes were performed. The 16S rRNA gene sequence was not sufficient to differentiate the bacteria within this group due to its high conservation. The nMLSA results were consistent with GBDP. Moreover, a fast typing method was proposed using the *pycA* gene, and where necessary, the *ccpA* gene. The pXO plasmids and *cry* genes were widely distributed, suggesting little correlation with the phylogenetic positions of the host bacteria. This might explain why classifications based on virulence characteristics proved unsatisfactory in the past. In summary, this is the first large-scale and systematic study of the taxonomic status of the bacteria within the *B. cereus* group using whole-genome sequences, and is likely to contribute to further insights into their pathogenicity, phylogeny and adaptation to diverse environments.

The *Bacillus cereus* group, also known as *B. cereus* sensu lato, consists of Gram-positive, rod-shaped, spore-forming aerobic bacteria that are widespread in natural environments. At the time of writing this manuscript, this group comprises eleven closely related species: *B. anthracis*, *B. cereus*, *B. thuringiensis*, *B. mycoides*, *B. pseudomycoides*, *B. weihenstephanensis*, *B. cytotoxicus*, *B. toyonensis*^1^, “*B. gaemokensis*”^2^, “*B. manliponensis*”^2^, and “*B. bingmayongensis*”^3^ (The names of the last three species are effectively but not yet validly published and thus are in quotation marks throughout this study). The former six species were identified during the 20th century, whereas the remaining five species were classified in recent years. Members of the *B. cereus* group have a significant impact on human health, agriculture, and the food industry^4^. For example, *B. anthracis* is the etiological agent of anthrax and an obligate pathogen that poses a threat to human and herbivore health, owing to the presence of two large plasmids, pXO1 and pXO2^5^. *B. cereus* is an opportunistic pathogen that often causes two forms of food poisoning, characterized by either nausea and vomiting or abdominal pain and diarrhea^6^. *B. thuringiensis* is an insect pathogen that is used worldwide in agriculture as a biopesticide based on the production of diverse crystal toxins^7^. In addition, the bacteria of the *B. cereus* group produce various valuable enzymes and metabolites^8^, degrade different types of pollutants and promote growth of both animals and plants when used as probiotics^9^,^10^.

In light of the significance of the *B. cereus* group, the identification and taxonomy of the isolates within the group are of fundamental importance, and therefore have been extensively studied using various typing methods from phenotype to genotype. In the past, the bacteria of this group were classified into different species according to 16S rRNA gene sequences and characteristics such as the presence or absence of virulence plasmids (*B. anthracis* and *B. thuringiensis*)^4^, colonial morphology (*B. mycoides* and *B. pseudomycoides*), psychrophilic or thermotolerant ability (*B. weihenstephanensis* and *B. cytotoxicus*)^9^,^11^, and other ill-defined features (*B. cereus*, vomiting- and diarrheal-type food poisoning)^6^. However, the group has been strikingly resistant to any type of satisfactory classification.

As mentioned above, the virulence plasmids play a critical role in discriminating the different species of this group relative to one another^4^, and to date still used as an important phenotypic feature owing to both scientific and social reasons. However, with the accumulation of exceptional strains, such a nomenclature based on the virulence plasmids is under great challenge^12^,^13^,^14^,^15^,^16^. For instance, strain *B. cereus* G9241 (numbered BCG14-02 in this study), isolated from a welder, contained pXO1 encoding all the primary virulence factors and really causing inhalational anthrax symptoms^12^. Likewise, strain *B. cereus* 03BB102 (numbered BCG01-30 in this study) harbored the pXO1^12^. On the contrary, some strains of *B. thuringiensis*, carrying insecticidal crystal protein genes (*cry*), are phylogenetically closely related to *B. anthracis*^14^,^16^. These results altered the belief that the virulence plasmids were found solely in *B. anthracis*, and poses a question to the accurate identification of *B. anthracis*. What exact phylogenetic position do these bacterial variants hold? Should they be named as *B. cereus* var. *anthracis*, as previously suggested^14^,^15^,^16^? Nevertheless, the classification based on virulence plasmids is evidently infeasible, as they can be easily transferred or lost in evolutionary history. Thus other methods should be assessed, and particularly a high-confidence phylogeny for the group is needed.

Recently, genotypic methods were commonly applied to differentiate members of this group. These approaches included analyses of single genes (i.e., *gyrB* and *plcR*)^17^,^18^, Amplified Fragment Length Polymorphism (AFLP)^19^, and traditional multi-locus sequence analysis (tMLSA)^18^,^20^,^21^,^22^ based on five to seven housekeeping genes. Although these approaches provided insights into the genetic diversity and phylogenetic relationships of the *B. cereus* group, they were still incapable of producing an accordant and convincing conclusion. Thus until now, the phylogenetic and taxonomic relationships of the bacteria of the *B. cereus* group are still under intense and controversial discussion^4^,^14^,^15^,^22^,^23^.DNA:DNA hybridization (DDH) values of 70% have been widely applied as a gold standard for the prokaryotic species definition^24^. With the advent of next-generation sequencing technologies, an increasing number of publicly available genome sequences has provoked a shift in the methods used for delineating bacterial species. Digital DNA:DNA hybridization (dDDH) is a fast and accurate replacement for the tedious and complicated traditional DDH^25^,^26^. This modern technique is based on the phylogenetically reliable^27^ Genome-Blast Distance Phylogeny method (GBDP)^25^,^26^,^28^,^29^ for calculating intergenomic distances, which are then converted to dDDH values. Digital DDH estimates, as delivered by the Genome-to-Genome Distance Calculator (GGDC), have been preferred over Average Nucleotide Identity (ANI) estimates because the GGDC provides higher correlations with traditional DDH results than do any of the ANI implementations^25^,^26^. The GGDC whole-genome approach has been successfully applied to many important genera, such as *Escherichia*^30^, *Hyphomonas*^31^ and *Thalassospira*^32^. Nucleotide GBDP is not only independent of gene calling and annotation but also provides a unique opportunity to obtain precise DDH estimates for species delimitation^26^ as well as phylogenies with statistical branch support^26^ in the same integrated approach. More recently, typing based on the chromosome via genomic comparison has been tried, but unfortunately it still cannot distinguish members of the *B. cereus* group from one another, most probably due to the inadequate genome data and insufficient methods^4^,^14^,^16^. At present, hundreds of genome sequences of the bacteria in the *B. cereus* group have been published. Thus, this provides an ideal opportunity for improving its taxonomy. A total of 224 genome sequences were used in this study, including all eleven type strains. A variety of approaches, including GBDP/dDDH analysis, 16S rRNA gene analysis, a specific-selection of genes for MLSA, and screening for virulence genes, were applied to (i) compare the outcomes of these methods, (ii) reconstruct the phylogeny of the bacteria of the *B. cereus* group, (iii) explore their genetic diversity, and (iv) obtain a more satisfactory classification.

## Result and Discussion

### Genome sequencing and data collection

A collection of 224 genomes of strains within the *B. cereus* group were used in this study, comprising 222 complete or draft genomes of *B. cereus* group isolates available in GenBank as well as two draft genomes of “*B. gaemokensis*” JCM 15801^T^ and “*B. manliponensis*” JCM 15802^T^, which were obtained by our laboratory. For each of the two type strains, a total of 500 megabases (Mb) of clean sequence data with an approximately 100-fold depth of coverage was generated for genome assembly by SOAPdenovo2. These two genome sequences were assembled into 256 and 101 contigs (>300 bp length), and deposited in GenBank under accession numbers JOTM00000000 and JOTN00000000, respectively. In Data S1, the main features of 224 genome sequences were summarized. The G+C content of the *B. cereus* group isolates ranged from 34.5 mol% (*B. thuringiensis* T01-328 and *B. thuringiensis* IBL 200) to 36.7 mol% (*B. cereus* Rock3-44), with a mean of 35.1 mol%. The genome sizes including plasmids, if any, varied from 4.09 Mb (*B. cytotoxicus* NVH 391-98^T^) to 7.09 Mb (*B. thuringiensis* T01-328), with an average of 5.77 Mb.

### Phylogenomic analyses

**Figure 1:**
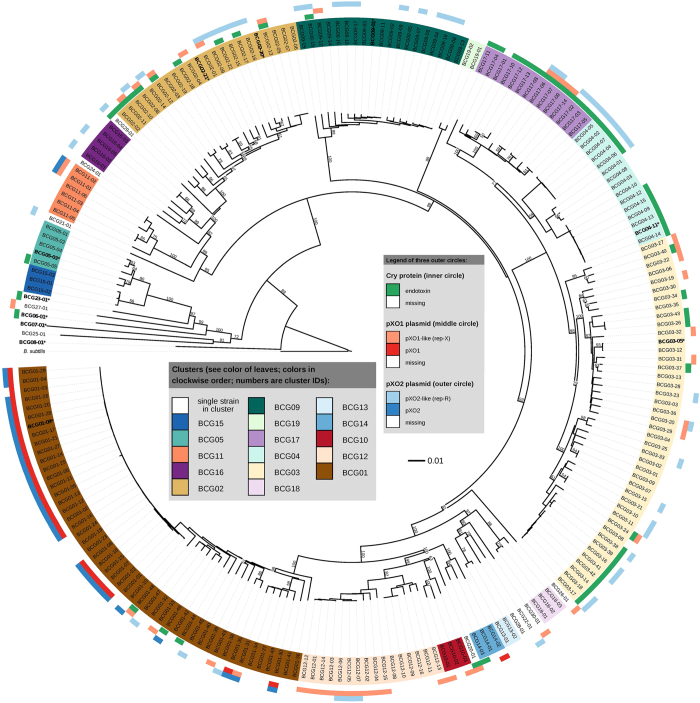
Whole-genome phylogeny of 224 bacteria of the *B. cereus* group inferred using the latest GBDP version and rooted with *Bacillus subtilis*. Numbers above branches are greedy-with-trimming pseudo-bootstrap^33^ support values from 100 replicates if larger than 50%. Leaves are colored according to their affiliation to clusters (i.e., *Bacillus cereus* groups, BCG). The three outer circles show whether or not the (i) *cry* locus, (ii) pXO1(-like) plasmid and/or the (iii) pXO2(-like) plasmid is found. Type strains are printed in bold font as well as marked by an asterisk (*). The tree was inferred using FastME^48^ and visualized using iTOL^57^. The leaf labels correspond to the encoding as listed in Data S1.

The pairwise dDDH estimates of the 225 genome sequences from 224 *B. cereus* strains, are listed in Data S2. The phylogenetic tree inferred from the pseudo-bootstrapped^26^ intergenomic distances (GGDs) is shown in Fig. 1 (the same tree with strain names included is shown in Figure S1) using *B. subtilis* subsp. *subtilis* ATCC 6051^T^ as an outgroup. The tree is well resolved, with most branches of the backbone receiving high branch support. As expected^30^,^33^, the greedy-with-trimming pseudo-bootstrapping yielded lower support values towards the leaves of the tree (Fig. 1). The clustering using a 70% dDDH radius around each of the eleven type strains yielded groups that occurred exactly in the linkage clustering with an *F* value of 0.5, which is shown in Fig. 1; the sole exception was the (non-)separation of *B. cereus* and *B. thuringiensis*, which is described in detail below.

The 224 strains were divided into 30 clusters, labelled BCG01 to BCG30, using the GGDC’s recommended-distance setting of 0.0361 (Fig. 1, Data S3); in contrast, they were split into 26 and 33 clusters according to two alternative distance thresholds of 0.0403 and 0.0322, respectively (Data S1). With few exceptions, the subtrees corresponding to these clusters received high to maximum support in the phylogenetic tree (Fig. 1). Ten of the 30 clusters (BCG01 - BCG09 and BCG23) contained 157 strains belonging to ten known species as indicated by the presence of the respective type strain. The other 20 clusters (BCG10 - BCG22 and BCG24 - BCG30) comprised 67 strains representing 20 potential novel species with the sole exception of cluster BCG17 that may also belong to *B. thuringiensis*, as described in detail below. Each cluster was documented below in details.

Cluster BCG01 contained 49 strains that could be further divided into several subclusters (Fig. 1, marked in 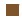). One was a highly conserved subcluster comprising 28 strains called *B. anthracis* in GenBank and the type strain *B. anthracis* A0465^T^ (BCG01-09). In contrast, the other subcluster showed a highly diverse branching pattern and consisted of 20 strains (BCG01-30 to BCG01-49) which had previously been identified as *B. cereus* or *B. thuringiensis* (and one strain only as a *Bacillus* sp.). These 20 strains belonged to *B. anthracis* based on the dDDH estimates. For the purpose of the following discussion, they were designated as anomalous *B. anthracis* strains to differentiate them from the traditional *B. anthracis* strains located in the first subcluster.

Cluster BCG02 accommodated 22 strains falling into two different species. The strains actually belonged to one species on the basis of the dDDH estimates and were recommended to be represented by the type strain *B. mycoides* DSM 2048^T^ (BCG02-20) (Fig. 1, marked in 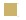). Among them, 19 strains were previously misidentified as *B. cereus* and should be reclassified as *B. mycoides*. The strain previously named as *B. weihenstephanensis* DSM 11821^T^ should be reclassified as *B. mycoides* as well, because they shared a high dDDH similarity of 78.2% ± 2.84 in contrast with a conventional DDH value of 86.9% (which nevertheless also indicate a single species) in a previous study^11^. Thus, *B. weihenstephanensis* should be considered as a later heterotypic synonym of *B. mycoides*.

The three closely related clusters BCG03, BCG04 and BCG17 included 63 strains and formed a clade in the tree, indicating that they share a common ancestor (Fig. 1). Cluster BCG03 was represented by *B. cereus* ATCC 14579^T^ (BCG03-05) and contained 34 strains defined previously as *B. cereus*, *B. thuringiensis* or *Bacillus* sp. Cluster BCG04 contained fifteen strains including the type strain *B. thuringiensis* ATCC 10792^T^ (BCG04-11). Cluster BCG17 comprised fourteen strains that were previously described as *B. cereus* or *B. thuringiensis*.

The separation of the strains to be assigned to *B. cereus* and those to be regarded as *B. thuringiensis* was less clear than between the other pairs of species. The dDDH between the two type strains was 71.2% ± 2.93 and thus slightly but not significantly above 70%. Nearly all of the 43 strains in cluster BCG03 yielded dDDH values ≥70% with the type strain of *B. cereus*, except two strains slightly below 70% (69.7% and 69.5%). The majority of them, 29 strains, also yielded dDDH values ≥70% with the type strain of *B. thuringiensis*. All 15 strains in cluster BCG04 yielded dDDH values ≥70% with the type strain of *B. thuringiensis*, but 10 of them also with the *B. cereus* type strain. The strains in cluster BCG17 yielded dDDH values clearly below 70% with all strains of BCG03 represented by the type strain of B. cereus. However, they yielded dDDH values not significantly smaller than 70% with most strains of BCG04 including the type strain of B. thuringiensis, even, they shared dDDH values slightly higher than 70% with other strains in BCG04. Thus a fusion of *B. cereus* and *B. thuringiensis* had the interesting consequence that not all of the strains with dDDH values ≥70% to at least one of two type strains also obtained dDDH ≥70% to the single relevant type strain that remained after merging the two species. It has recently been emphasized that such discrepancies are caused by non-ultrametricity (e.g., due to the deviation from a molecular clock^34^) and can occur with all kinds of uses of pairwise distances or similarities to directly draw taxonomic conclusions, not only with traditional DDH or dDDH^35^. Fortunately, non-ultrametricity need not cause problems for a certain data set for a given threshold because it rarely occurs in GGDC-based taxonomic analysis^35^, and in the current study, it only affected the distinction between *B. cereus* and *B. thuringiensis*. For reasons of taxonomic conservatism, we suggest regarding the two species as separate, and for now assigning BCG17 to *B. thuringiensis*. Taxonomists who preferred to merge *B. cereus* and *B. thuringiensis* would need to regard them as heterotypic synonyms; no formal taxonomic changes would need to be proposed^36^. Based on the analysis of virulence genes described below, differences in the plasmid distribution between the clusters BCG03 and BCG04 were indicated in Fig. 1. The occurrence of plasmids seemed to greatly vary according to the specific subgroup of either cluster and thus could not provide a clear differentiation.

In addition to the five clusters mentioned above, the remaining 25 clusters consisted of 90 strains corresponding to six well-defined species and 19 putative novel species. The six clusters were formed by 28 strains, including clusters BCG05, BCG06, BCG07, BCG08, BCG09 and BCG23, which corresponded to the species *B. pseudomycoides*, “*B. gaemokensis*”, “*B. manliponensis*”, *B. cytotoxicus*, *B. toyonensis* and “*B. bingmayongensis*”, respectively. The remaining 19 new clusters each represented a putative novel species. Among these clusters, cluster BCG11, cluster BCG12 and cluster BCG16 contained six, sixteen and five strains, respectively and were quite diverse. In contrast, the remaining 16 clusters contained no more than three, respectively. These small subgroups were scattered between other clusters or were deeply branching according to Fig. 1, indicating an early differentiation within the *B. cereus* group.

Prior to this study, multiple approaches were applied to distinguish between the strains within the *B. cereus* group^16^,^17^,^19^,^20^,^22^. Disappointingly, an ever-increasing number of investigations were incapable of providing an accurate and consistent classification within this group. For example, numerous studies suggested that the three most important species, *B. anthracis*, *B. cereus* and *B. thuringiensis*, should be considered a single species^23^,^37^, whereas other studies observed sufficient genetic differentiation between these strains to suggest three distinct species^38^,^39^. At present, a more widely accepted viewpoint is that the *B. cereus* group comprises seven major phylogenetic groups (I to VII)^40^, but these groups only partially agree with our genome-scale results. It was amazing and unsettling that, 149 of 224 genomes deposited in GenBank were misidentified at the species level according to the GBDP results, thereby yielding misnomers that could easily be propagated in other databases and affect subsequent studies. The taxonomic controversies of strains within the *B. cereus* group were likely caused, at least in part, by imperfections in past and current methods that hindered the ability of the taxonomist to consistently distinguish between these strains. These limitations included the high conservation of the 16S rRNA gene sequences compared with its intra-strain diversity as described below, the lack of reproducibility of DNA fingerprinting results and their consistency between laboratories^38^, and the conflicting signals from horizontal gene transfer (HGT) and recombination events in tMLSA^32^. These disagreements highlight the vital importance of genomic typing in taxonomy.

### The 16S rRNA gene sequence analysis

**Figure 2:**
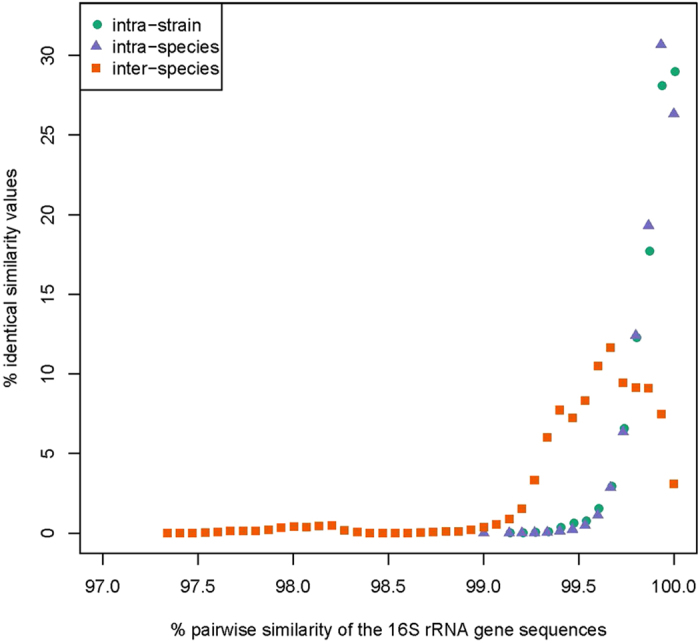
Distribution of pairwise 16S rRNA gene similarities of the three levels: inter-species, intra-species and intra-strain. A total of 1007 16S rRNA gene sequences were analyzed, sharing all sites from positions 352 to 1051 in the complete 16S rRNA gene; more sequences could not be considered due to the condition of some of the draft genome sequences. The x-axis indicated the pairwise similarity (in %) of the 16S rRNA gene sequences, whereas the y-axis represents the proportion of each respective similarity value.

In general, the 16S rRNA gene plays an important role in microbial identification and taxonomy. Nevertheless, its suitability for the classification of the *B. cereus* group might be limited, and its genomic distribution in that group is still not fully understood. Therefore, the distribution of 16S rRNA operons from the 224 isolates of the *B. cereus* group was first investigated. A total of 1449 16S rRNA gene sequences were obtained from 223 strains of the *B. cereus* group (Data S1), with the exception of *B. anthracis* A2012, whose draft genome sequence lacked RNA operons^41^. We found that 16S rRNA gene copy numbers per strain ranged from one to 19 with 6.5 operons on average. The copy number variation, especially in strains with low numbers, may have resulted from incomplete genome sequencing and/or an overlap of the same 16S rRNA gene during splicing. When considering only the completed genomes, 16S rRNA gene copy numbers ranged between 10 and 15. The high copy number of 16S rRNA gene in our study is in accordance with reports for *Firmicutes*^42^. Secondly, pairwise similarities between 16S rRNA gene sequences were calculated, and related to the dDDH/GGD clusters described above, each representing a putative species. As shown in Fig. 2, the 16S rRNA divergence levels overlapped between inter- (97.34–100%) and intra-species comparisons (99.14–100%). The results explicitly indicate that the resolution of the 16S rRNA gene is too low to discriminate closely related species^43^. Similar cases were often encountered in other groups of closely related species within genera such as *Aeromonas*^44^, *Pseudomonas*^45^ and *Vibrio*^46^.

A signature sequence of the 16S rRNA gene (^1008^**T**CTAGAGATAG**A**) was suggested to be characteristic of *B. weihenstephanensis*^11^. However, in this study, we found that this signature sequence also occurred in some clusters. Moreover, no other signature sequence could be detected for the inferred dDDH/GBDP clusters. All sequences at the intra-strain, inter-strain and intra-species levels in the phylogenetic tree of 16S rRNA gene (Figure S2) exhibited only partially accurate clustering, and with low bootstrap support values, compared to the dDDH/GBDP analysis.

We conclude that the 16S rRNA gene is unable to effectively distinguish between the closely related species within the *B. cereus* group, in agreement with previous results^47^.

### MLSA phylogenetic analysis

**Figure 3:**
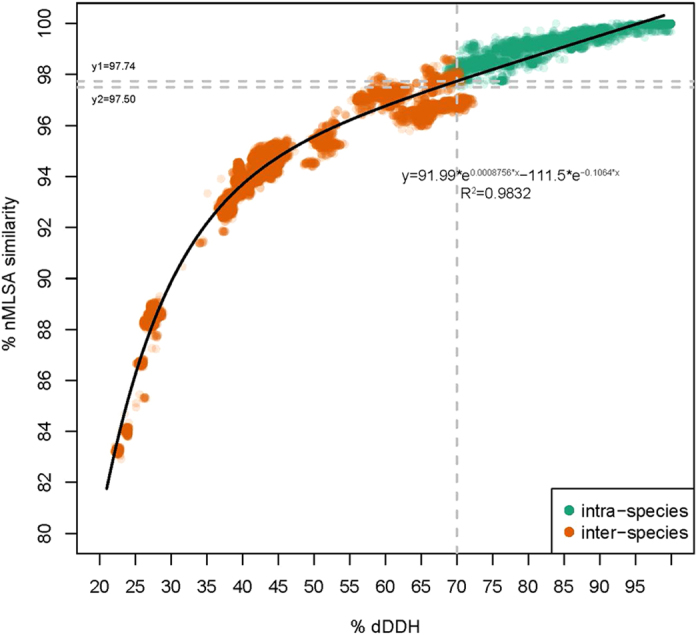
Correlation analysis between dDDH values and nMLSA similarities. The exponential equation (y = 91.99*e^0.0008756*x^ – 111.5*e^−0.1064*x^, R^2^ = 0.9832) was obtained using a nonlinear simulation analysis method with the default option of the Curve Fitting Tool implemented in MATLAB 8.1. The vertical line indicates the 70% dDDH threshold. The upper horizontal line (y1 = 97.74) indicates the estimated nMLSA similarity threshold (inter-species) corresponding to 70% dDDH threshold that was calculated on the basis of the above exponential equation. The lower horizontal line (y1 = 97.50) indicates the actual lowest similarity for nMLSA among strains of intra-species.

In recent years, tMLSA approaches have been developed and widely used to discriminate between strains of the *B. cereus* group^18^,^20^,^21^,^22^. Despite their higher resolution, these strategies failed at developing a standardized typing scheme and at yielding consistent results. Thus, a more powerful method needs to be established. In this report, the novel MLSA (nMLSA) scheme based on a concatenation of 20 housekeeping genes was performed to discern the phylogenetic relationships of 224 strains within the *B. cereus* group. The nMLSA phylogenetic tree is shown in Figure S3; the tree is topologically congruent with the GBDP phylogeny, particularly regarding the well-supported branches (Figure S3). Based on nMLSA, 224 strains could be divided into 30 clusters representing the 29–30 (known or putative new) species determined in the GBDP analysis. Furthermore, these clusters were supported by high bootstrap values in nMLSA analysis. The nMLSA similarities revealed a strong correlation with the dDDH values (R^2^ = 0.9832). Moreover, the 70% dDDH threshold for species definition corresponded to 97.74% similarity in nMLSA, based on the simulative exponential equation (y = 91.99*e^0.0008756*x^ – 111.5*e^−0.1064*x^) (Fig. 3). Nevertheless, as shown in Fig. 3, strains sharing an nMLSA similarity greater than 97.74% were not guaranteed to belong to the same species. This discrepancy might be caused by non-ultrametricity in the nMLSA data^48^. The relationship between DDH and sequence divergence in tMLSA was also widely reported in other genera such as *Aeromonas*^49^, *Thalassospira*^32^ and *Vibrio*^50^, but the threshold value for species definition in tMLSA varied across taxa^51^,^52^.

**Figure 4:**
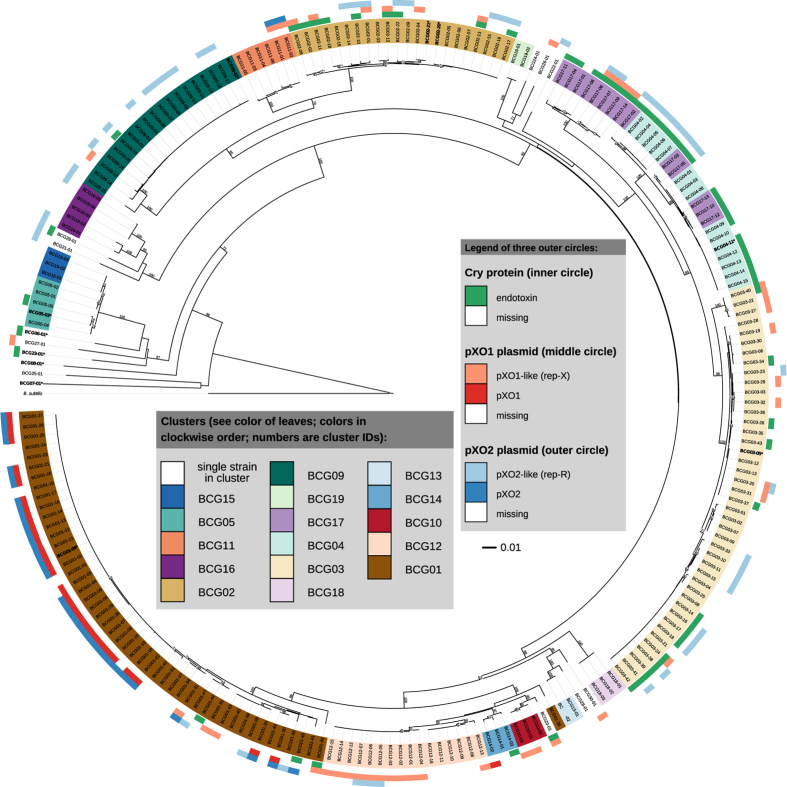
Phylogenetic trees inferred from the *pycA* gene of 224 bacteria of the *B. cereus* group. Numbers on branches are bootstrap support values from 1,000 replicates given in percent. Branches are scaled in terms of the expected number of substitutions per site. Leaves are colored according to their affiliation to clusters (compare Fig. 1). The three outer circles show whether or not the (i) *cry* locus, (ii) pXO1(-like) plasmid and/or the (iii) pXO2(-like) plasmid are found. Type strains are printed in bold font and marked by an asterisk (*). *Bacillus subtilis* ATCC 6051^T^ (CP003329) was used as the outgroup.

With the rapid improvement of sequencing technology and bioinformatic tools, bacterial taxonomy for delineating species has become more feasible and efficient. However, both nMLSA and dDDH could currently still be rather impractical in view of the genome-sequencing efforts, the required know-how and the costs necessary for a rapid identification of a large number of strains. We thus tried to find an alternative by screening some of the robust marker genes used in species discrimination. As illustrated in Fig. 4, the *pycA* gene could rapidly differentiate strains from 26 of 30 different clusters with the exception of two paired clusters BCG04/BCG17 and BCG05/BCG15. In these cases, the *ccpA* gene could be used to distinguish between strains from each pair of clusters, respectively (Figure S4). Therefore, the *pycA* gene and, where necessary, the *ccpA* gene are proposed for the fast identification of isolates within the *B. cereus* group.

### The distribution of virulence genes among the isolates of this group

Within the *B. cereus* group, the virulence plasmids are closely linked to disease symptoms and host specificity^6^,^7^. They have been widely applied to separate *B. anthracis*, *B. cereus* and *B. thuringiensis*^4^,^17^. In this study, the pXO plasmids and *cry* genes were identified throughout the genomes of 224 strains using the criteria for the presence of virulence plasmids described in ‘Materials and Methods’ (Data S1, Fig. 1). The pXO1 and pXO2 plasmids were primarily harboured in the bacteria in cluster BCG01, especially the traditional *B. anthracis* which were highly conserved. pXO1 was identified in 26 *B. anthracis* strains of cluster BCG01 and strain BCG14-02 within the putative novel cluster BCG14. A total of 27 *B. anthracis* strains harboured the pXO2 plasmid as well as another two strains of BCG11-01 and BCG11-02 belonging to the potential novel cluster BCG11. Thus, pXO1 and pXO2 only coexisted in *B. anthracis* strains, including 23 traditional and two anomalous ones. However, the pXO-like plasmids are widespread in many other bacteria, and are expected to be subject to certain selection pressures exercised by the host (Fig. 1). For example, 43 strains from twelve distinct species contained the pXO1-like plasmid, which occurred more frequently in strains of several clusters close to *B. anthracis*; in contrast, 50 strains from eleven species contained a pXO2-like plasmid, which was more widespread among the non-*B. anthracis* species and seldom co-occurred with the pXO1-like. Moreover, the pXO2-like plasmid more frequently occurred in strains of cluster BCG09.

Traditional *B. thuringiensis* strains usually contain *cry* genes that encode large Cry protein inclusions of the *δ*-endotoxin. Indeed, crystal formation is considered a typical feature of *B. thuringiensis* bacteria^6^,^16^. We found, however, that the species containing the *cry* gene were quite diverse in this group. The *cry* genes were identified in 57 strains from twelve clusters (Fig. 1); they were most common in BCG04 and BCG17 clusters. Thus, the presence or absence of *cry* genes cannot be used to discriminate between *B. cereus* and *B*. *thuringiensis*.

Overall, the presence or absence of the virulence plasmids and the *cry* gene is largely uncorrelated with the phylogenetic position of the host bacteria. These results are in agreement with those of recent studies^14^,^15^,^16^. Previous taxonomic disorder of the *B. cereus* group is largely due to inadequate criteria based on virulence characteristics, which are residing on virulence plasmids.

## Conclusion

In this study, the evolutionary relationships within the *B. cereus* group were resolved using genome-sequence analysis of 224 strains. We conducted the first large-scale, whole-genome sequence-based systematic study of the phylogenetic affiliations within this group. The results demonstrate that the *B. cereus* group can be divided into 30 clusters, each representing independent species including 19–20 putative novel species in addition to eleven previously described species, two of which have to be merged. The dispute concerning *B. anthracis*, *B*. cereus and *B. thuringiensis* can be resolved by the systematic analysis of whole genome sequences. The housekeeping genes *pycA* and, where necessary, *ccpA* are recommended for the fast identification of any isolate of this group. In contrast, the toxic gene-carrying plasmids such as pXO in *B. anthracis* and the *cry* gene plasmids in *B. thuringiensis* cannot serve as signatures of either species. Indeed, using dDDH, some strains previously identified as *B. cereus* or *B. thuringiensis* were identified as *B. anthracis*. These bacteria should be paid much more attention to re-evaluate their biosafety, especially the bacteria carrying *cry* genes when used as biopesticides.

In addition to strains traditionally ascribed to *B. anthracis*, that were highly conserved and toxic, others that were quite diverse should be included in the species according to the 70% dDDH criterion. Some of these anomalous *B. anthracis* strains are also a potential threat to human health due to the presence of pXO1 or pXO2, or the possible reception of exogenous virulence plasmids. The pXO1 and pXO2 mainly coexist within the monophyletic group of traditional *B. anthracis* with only a few exceptions. This result, derived from the whole-genome data, might indicate that traditional, highly conserved and highly pathogenic *B. anthracis* bacteria have specifically adapted to humans and herbivorous mammals, possibly via the co-evolution of the chromosomes and virulence plasmids. But how *B. anthracis* has differentiated as a separate species still remains an open question.

In summary, in this report, a large-scale, systematic and phylogenomic study of the controversial *B. cereus* group was performed. Two species were recognized as heterotypic synonyms, while 19–20 novel species were revealed and await further characterization. The results of this study clarify the entangled nomenclature of these bacteria and provide an avenue to a better understanding of pathogenicity, ecological role and evolutionary relationships of the strains of this group.

## Materials and Methods

### Genome sequence data

In this study, 224 available genomes sequences from the *B. cereus* group were utilized (Data S1). To be specific, 222 genome sequences were obtained from the National Center for Biotechnology Information (NCBI) database. Two type strains, “*B. gaemokensis*” JCM 15801^T^ and “*B. manliponensis*” JCM 15802^T^, were purchased from the Japan Collection of Microorganisms (JCM). Their genome sequences were determined using Solexa paired-end sequencing technology by Shanghai Majorbio Bio-Pharm Technology Co., Ltd. (Shanghai, China). All 224 genome sequences were (re-)annotated with the Rapid Annotation of microbial genomes using Subsystems Technology (RAST) version 4.0 (http://rast.nmpdr.org/)^53^. *B. subtilis* (type species of the genus) ATCC 6051^T^ (CP003329) was used as an outgroup in all phylogenetic analyses.

### Assessment of dDDH and further phylogenomic analysis

The 70% DDH threshold and analogous genome-sequence based methods are still the taxonomist’s main criterion for assessing species affiliation^24^. Instead of using the tedious traditional approach, we here used the digital variant (dDDH) of the method. It yields the highest^26^ consistency regarding the microbial species concept and at the same time avoids the pitfalls of traditional DDH due to the much lower error rate in genome sequencing^35^. Hence, all pairwise dDDH estimates between 225 strains were obtained *via* the GGDC 2.0, under the recommended Formula 2^25^,^26^. Since dDDH implemented in GGDC is based on GGDs calculated with the GBDP^28^,^29^, these GGDs could be used to infer a phylogenetic tree with FastME^48^ including branch-support values^26^. Estimates for species affiliations were obtained by clustering the GGDs with the distance threshold corresponding to 70% dDDH (0.0361 for the recommended GGDC setting) and non-hierarchical linkage clustering with an *F* value of 0.5 as implemented in OTPSIL^54^. This *F* value yielded the highest clustering consistency for the present data at the predefined threshold, similar to other scenarios^30^. Then, the clustering was compared to the clustering implied by the 70% dDDH environments of each type strain^35^. Stability of the clustering was assessed by using the distances (0.0322, 0.0403) whose lower or upper confidence boundaries^26^ corresponded to 70% dDDH as alternative thresholds.

### The 16S rRNA gene and nMLSA

The 16S rRNA gene sequences and other gene sequences were obtained from 225 genome sequences using BLASTN. A total of 233 single-copy genes belonging to the minimal bacterial gene set required to sustain a living cell^55^ were considered as candidates for nMLSA. In total, 20 genes (listed in Data S3) were selected from the candidate genes on the basis of relatively high resolution power. All gene sequences were aligned using ClustalW in MEGA version 5.0^56^. The alignments were trimmed to match the length of the shortest contained open reading frame. The nMLSA was performed using 20 concatenated gene sequences, with the phylogenetic reconstruction done under the Kimura’s 2-parameter model in conjunction with the neighbour-joining (NJ) method, as implemented in MEGA version 5.0^56^. The reliability of each tree topology was assessed using 1,000 bootstrap replicates. Pairwise gene similarities were calculated via p-distances. The relationship between dDDH values and nMLSA similarities was determined using a nonlinear simulation analysis method with the default option of the Curve Fitting Tool implemented in MATLAB 8.1. The visualization and annotation of all phylogenetic trees was performed using the web-based tool Interactive Tree Of Life (iTOL)^57^.

### Screening of the virulence genes in plasmids

The screening of virulence genes in plasmids was performed using a combined analysis of the RAST^53^ annotation and local BLASTN in the 224 genomes. Briefly, the most characteristic genes (Data S4) from *B. anthracis* Ames Ancestor, including four genes of the pXO1 plasmid (*cya*, *lef*, *pagA* and *repX*)^58^ and six genes of the pXO2 plasmid (*capA*, *capB*, *capC*, *capD*, *capE* and *repS*)^59^, were used as the reference sequences. The BLASTN threshold for both similarity and coverage was 30%, and all BLAST results were cross-checked against the RAST annotation. The criteria for the presence of virulence plasmids were established as follows. If all four pXO1 genes were discovered, we assumed that the isolate contained the pXO1 plasmid, whereas the presence of only one to three genes indicated the presence of a pXO1-like plasmid. Similarly, if all six pXO2 genes were found, we assumed the presence of the pXO2 plasmid, while the determination of one to five genes suggested the existence of a pXO2-like plasmid. The *cry* genes were detected using the BtToxin_scanner website (http://bcam.hzaubmb.org/BtToxin_scanner/)^60^.

## Additional Information

**Accession code:** Two draft genome sequences “*B. gaemokensis*” JCM 15801^T^ and “*B. manliponensis*” JCM 15802^T^ were deposited in GenBank under accession numbers JOTM00000000 and JOTN00000000, respectively.

**How to cite this article**: Liu, Y. *et al*. Genomic insights into the taxonomic status of the *Bacillus cereus* group. *Sci. Rep*. **5**, 14082; doi: 10.1038/srep14082 (2015).

## Supplementary Material

Supplementary Information

Supplementary Table S5
