# Supplementary material for: Genomic insights into the taxonomic status of the Bacillus cereus group
Source: Sci Rep. 2015 Sep 16;5:14082. doi: 10.1038/srep14082 (PMC4571650; doi:10.1038/srep14082)
Supplement: Supplementary Information [file srep14082-s1.pdf]

Genomic insights into the taxonomic status of the *Bacillus cereus* group

Yang Liu[1†], Qiliang Lai[1†], Markus Göker[2†], Jan P. Meier-Kolthoff[2†], Meng Wang[3], Yamin Sun[3], Lei Wang[3] and Zongze Shao[1*]

[1]State Key Laboratory Breeding Base of Marine Genetic Resources; Key Laboratory of Marine Genetic Resources, Third Institute of Oceanography, SOA; South China Sea Bio-Resource Exploitation and Utilization Collaborative Innovation Centre; Fujian Collaborative Innovation Center for Exploitation and Utilization of Marine Biological Resources; Key Laboratory of Marine Genetic Resources of Fujian Province, Xiamen 361005, China

[2]Leibniz Institute DSMZ – German Collection of Microorganisms and Cell Cultures GmbH, Inhoffenstraße 7B, 38124, Braunschweig, Germany

[3]TEDA School of Biological Sciences and Biotechnology Nankai University, Tianjin, China

*Corresponding author: Zongze Shao. Tel: +86-592-2195390. Fax: +86-592-2085376.

E-mail: shaozz@163.com

[†] **co-first author**

Contents

Supplementary Figures 3

Figure S1 | Whole-genome phylogeny of 224 bacteria of the *B. cereus* group inferred using the latest GBDP version and rooted with *Bacillus subtilis* ATCC 6051^{T} 4

Figure S2 | Phylogenetic tree inferred from 1007 copies of the 16S rRNA gene of 202 bacteria of the *B. cereus* group 14

Figure S3 | nMLSA-based phylogenetic tree of 224 bacteria of the *B. cereus* group using 20 housekeeping genes 16

Figure S4 | Phylogenetic tree inferred from the *ccpA* gene of 224 bacteria of the *B. cereus* group 18

Supplementary Data

Data S1 | Complete list of the 225 strains used in this study with detailed annotations

Data S2 | Pairwise dDDH values between the 224 strains of the *B. cereus* group

Data S3 | Distribution of 224 strains into 30 clusters (putative species)

Data S4 | The 20 housekeeping genes used for the nMLSA analysis

Data S5 | List of characteristic genes used as reference sequences for the local Blastn

Supplementary Figures

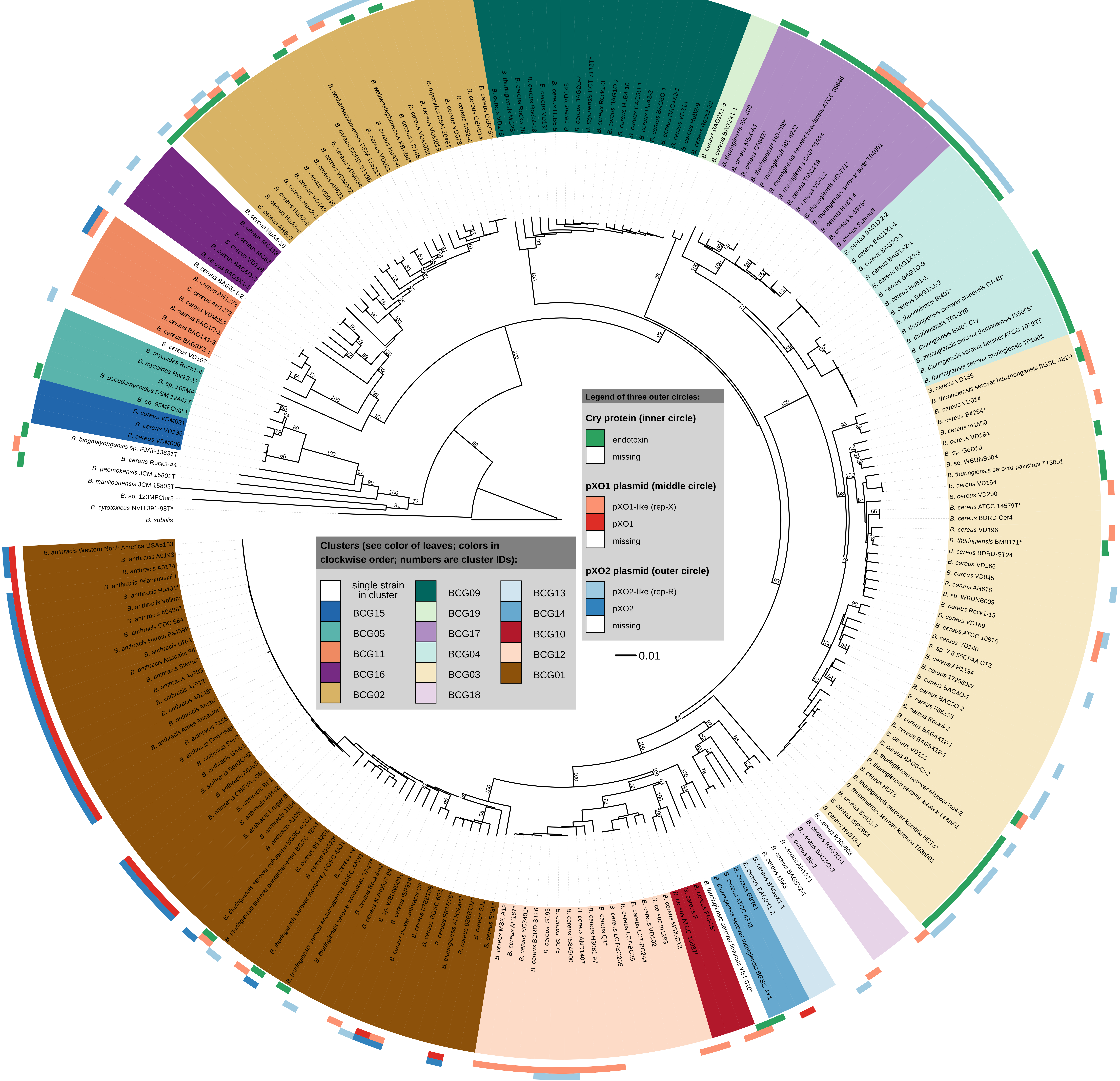

Figure S1 | Whole-genome phylogeny of 224 bacteria of the *B. cereus* group inferred using the latest GBDP version and rooted with *Bacillus subtilis* ATCC 6051^{T}. Numbers above branches are greedy-with-trimming pseudo-bootstrap support values from 100 replicates if larger than 50%. Leaves are colored according to their affiliation to clusters. The three outer circles show whether or not the (i) *cry* locus, (ii) pXO1(-like) plasmid and/or the (iii) pXO2(-like) plasmid is found. Type strains are printed with "T". The strains with a complete genome sequence are marked by an asterisk (*). The tree was inferred using FastME and visualized using iTOL. The leaf labels are strain names according to the NCBI database. Please note that the species names "*B. gaemokensis*", "*B. manliponensis*" and *"B. bingmayongensis*" are effectively but not yet validly published.

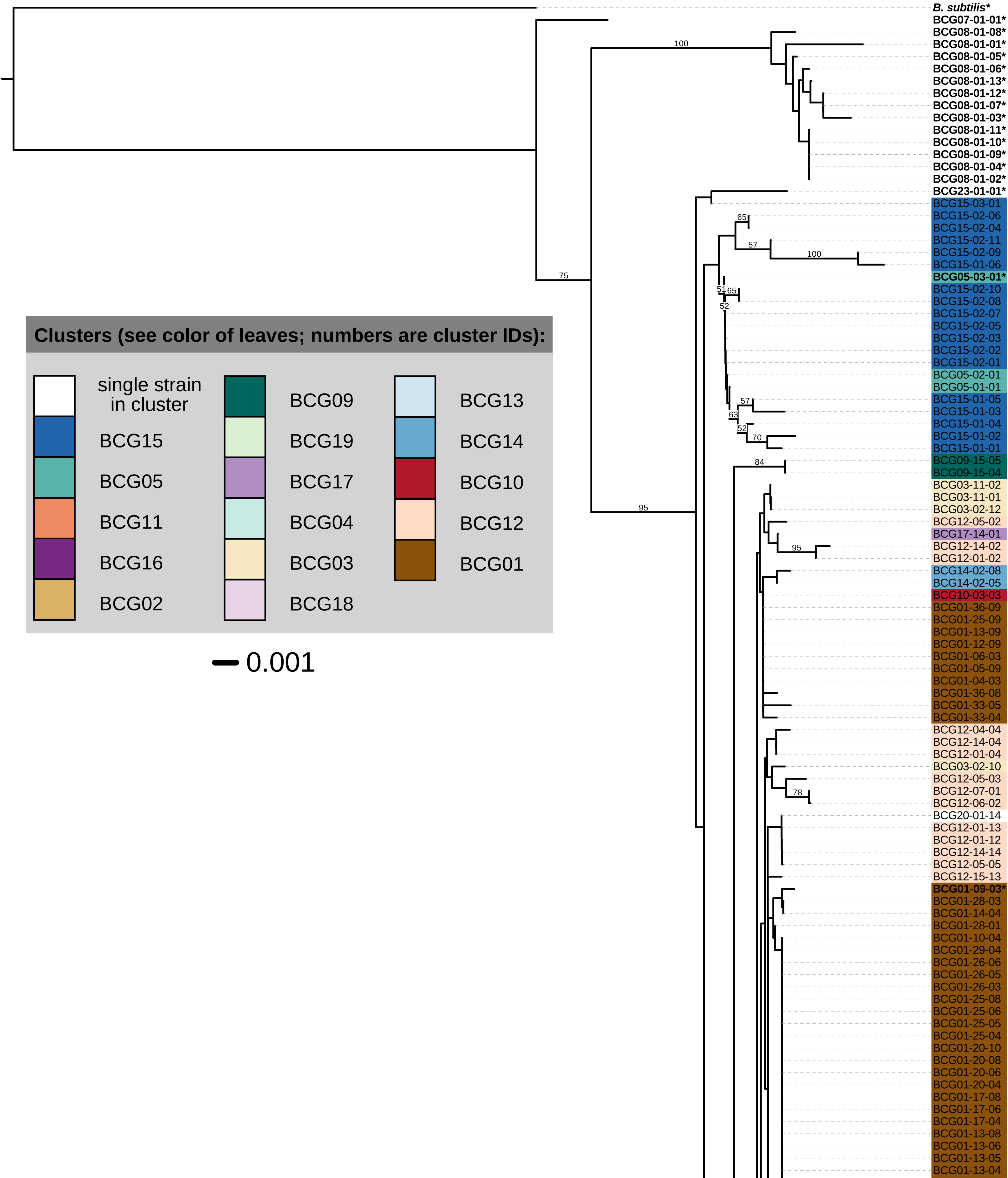

B. subtilis*
BCG07-01-01*
BCG08-01-08*
BCG08-01-01*
BCG08-01-05*
BCG08-01-06*
BCG08-01-13*
BCG08-01-12*
BCG08-01-07*
BCG08-01-03*
BCG08-01-11*
BCG08-01-10*
BCG08-01-09*
BCG08-01-04*
BCG08-01-02*
BCG23-01-01*
BCG15-03-01
BCG15-02-06
BCG15-02-04
BCG15-02-11
BCG15-02-09
BCG15-01-06
BCG05-03-01*
BCG15-02-10
BCG15-02-08
BCG15-02-07
BCG15-02-05
BCG15-02-03
BCG15-02-02
BCG15-02-01
BCG05-02-01
BCG05-01-01
BCG15-01-05
BCG15-01-03
BCG15-01-04
BCG15-01-02
BCG15-01-01
BCG09-15-05
BCG09-15-04
BCG03-11-02
BCG03-11-01
BCG03-02-12
BCG12-05-02
BCG17-14-01
BCG12-14-02
BCG12-01-02
BCG14-02-08
BCG14-02-05
BCG10-03-03
BCG01-36-09
BCG01-25-09
BCG01-13-09
BCG01-12-09
BCG01-06-03
BCG01-05-09
BCG01-04-03
BCG01-36-08
BCG01-33-05
BCG01-33-04
BCG12-04-04
BCG12-14-04
BCG12-01-04
BCG03-02-10
BCG12-05-03
BCG12-07-01
BCG12-06-02
BCG20-01-14
BCG12-01-13
BCG12-01-12
BCG12-14-14
BCG12-05-05
BCG12-15-13
BCG01-09-03*
BCG01-28-03
BCG01-14-04
BCG01-28-01
BCG01-10-04
BCG01-29-04
BCG01-26-06
BCG01-26-05
BCG01-26-03
BCG01-25-08
BCG01-25-06
BCG01-25-05
BCG01-25-04
BCG01-20-10
BCG01-20-08
BCG01-20-06
BCG01-20-04
BCG01-17-08
BCG01-17-06
BCG01-17-04
BCG01-13-08
BCG01-13-06
BCG01-13-05
100
75
95
65
57
100
51
65
52
57
63
52
70
84
95
78
Clusters (see color of leaves; numbers are cluster IDs):
single strain in cluster
BCG15
BCG05
BCG11
BCG16
BCG02
BCG09
BCG19
BCG17
BCG04
BCG03
BCG18
BCG13
BCG14
BCG10
BCG12
BCG01
0.001

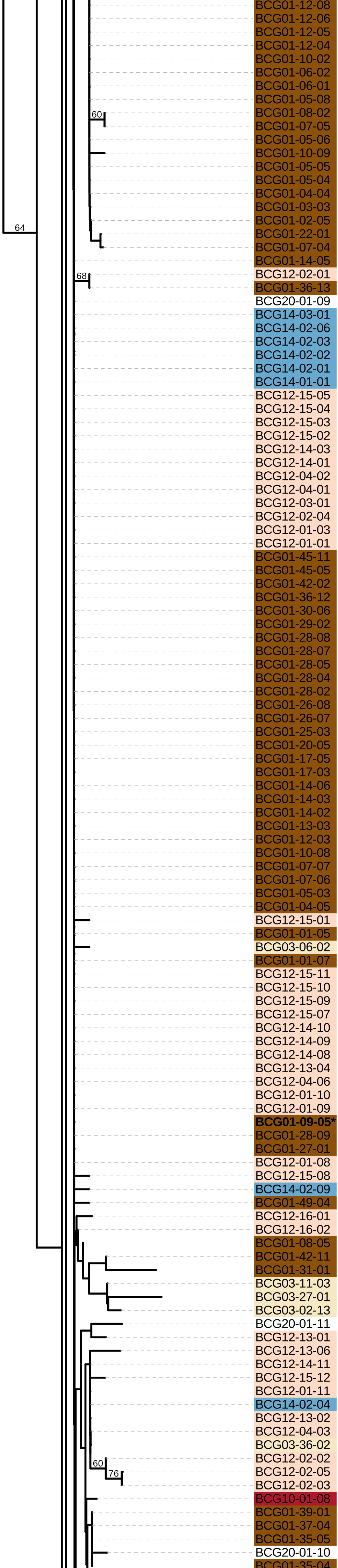
60
64
68
60
76
BCG01-12-08
BCG01-12-06
BCG01-12-05
BCG01-12-04
BCG01-10-02
BCG01-06-02
BCG01-06-01
BCG01-05-08
BCG01-08-02
BCG01-07-05
BCG01-05-06
BCG01-10-09
BCG01-05-05
BCG01-05-04
BCG01-04-04
BCG01-03-03
BCG01-02-05
BCG01-22-01
BCG01-07-04
BCG01-14-05
BCG12-02-01
BCG01-36-13
BCG20-01-09
BCG14-03-01
BCG14-02-06
BCG14-02-03
BCG14-02-02
BCG14-02-01
BCG14-01-01
BCG12-15-05
BCG12-15-04
BCG12-15-03
BCG12-15-02
BCG12-14-03
BCG12-14-01
BCG12-04-02
BCG12-04-01
BCG12-03-01
BCG12-02-04
BCG12-01-03
BCG12-01-01
BCG01-45-11
BCG01-45-05
BCG01-42-02
BCG01-36-12
BCG01-30-06
BCG01-29-02
BCG01-28-08
BCG01-28-07
BCG01-28-05
BCG01-28-04
BCG01-28-02
BCG01-26-08
BCG01-26-07
BCG01-25-03
BCG01-20-05
BCG01-17-05
BCG01-17-03
BCG01-14-06
BCG01-14-03
BCG01-14-02
BCG01-13-03
BCG01-12-03
BCG01-10-08
BCG01-07-07
BCG01-07-06
BCG01-05-03
BCG01-04-05
BCG12-15-01
BCG01-01-05
BCG03-06-02
BCG01-01-07
BCG12-15-11
BCG12-15-10
BCG12-15-09
BCG12-15-07
BCG12-14-10
BCG12-14-09
BCG12-14-08
BCG12-13-04
BCG12-04-06
BCG12-01-10
BCG12-01-09
BCG01-09-05*
BCG01-28-09
BCG01-27-01
BCG12-01-08
BCG12-15-08
BCG14-02-09
BCG01-49-04
BCG12-16-01
BCG12-16-02
BCG01-08-05
BCG01-42-11
BCG01-31-01
BCG03-11-03
BCG03-27-01
BCG03-02-13
BCG20-01-11
BCG12-13-01
BCG12-13-06
BCG12-14-11
BCG12-15-12
BCG12-01-11
BCG14-02-04
BCG12-13-02
BCG12-04-03
BCG03-36-02
BCG12-02-02
BCG12-02-05
BCG12-02-03
BCG10-01-08
BCG01-39-01
BCG01-37-04
BCG01-35-05
BCG20-01-10
BCG01-35-04

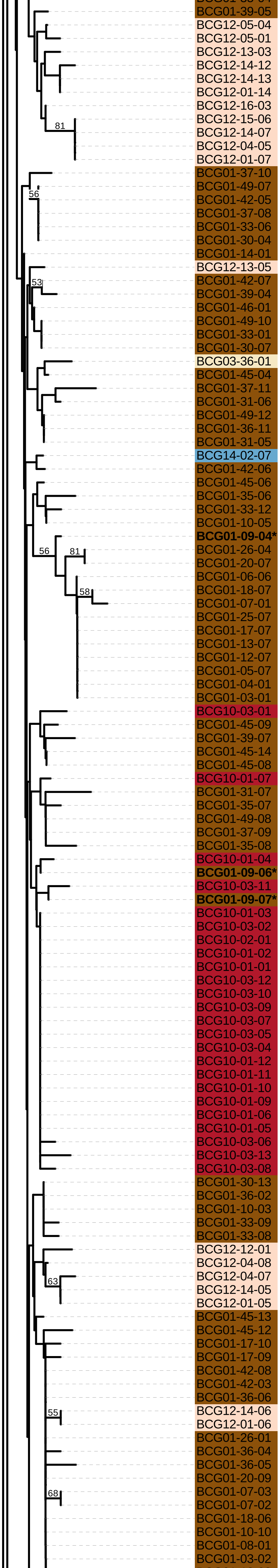
BCG01-39-05
BCG12-05-04
BCG12-05-01
BCG12-13-03
BCG12-14-12
BCG12-14-13
BCG12-01-14
BCG12-16-03
BCG12-15-06
81
BCG12-14-07
BCG12-04-05
BCG12-01-07
BCG01-37-10
BCG01-49-07
56
BCG01-42-05
BCG01-37-08
BCG01-33-06
BCG01-30-04
BCG01-14-01
BCG12-13-05
53
BCG01-42-07
BCG01-39-04
BCG01-46-01
BCG01-49-10
BCG01-33-01
BCG01-30-07
BCG03-36-01
BCG01-45-04
BCG01-37-11
BCG01-31-06
BCG01-49-12
BCG01-36-11
BCG01-31-05
BCG14-02-07
BCG01-42-06
BCG01-45-06
BCG01-35-06
BCG01-33-12
BCG01-10-05
BCG01-09-04*
56
81
BCG01-26-04
BCG01-20-07
BCG01-06-06
58
BCG01-18-07
BCG01-07-01
BCG01-25-07
BCG01-17-07
BCG01-13-07
BCG01-12-07
BCG01-05-07
BCG01-04-01
BCG01-03-01
BCG10-03-01
BCG01-45-09
BCG01-39-07
BCG01-45-14
BCG01-45-08
BCG10-01-07
BCG01-31-07
BCG01-35-07
BCG01-49-08
BCG01-37-09
BCG01-35-08
BCG10-01-04
BCG01-09-06*
BCG10-03-11
BCG01-09-07*
BCG10-01-03
BCG10-03-02
BCG10-02-01
BCG10-01-02
BCG10-01-01
BCG10-03-12
BCG10-03-10
BCG10-03-09
BCG10-03-07
BCG10-03-05
BCG10-03-04
BCG10-01-12
BCG10-01-11
BCG10-01-10
BCG10-01-09
BCG10-01-06
BCG10-01-05
BCG10-03-06
BCG10-03-13
BCG10-03-08
BCG01-30-13
BCG01-36-02
BCG01-10-03
BCG01-33-09
BCG01-33-08
BCG12-12-01
BCG12-04-08
63
BCG12-04-07
BCG12-14-05
BCG12-01-05
BCG01-45-13
BCG01-45-12
BCG01-17-10
BCG01-17-09
BCG01-42-08
BCG01-42-03
BCG01-36-06
55
BCG12-14-06
BCG12-01-06
BCG01-26-01
BCG01-36-04
BCG01-36-05
BCG01-20-09
68
BCG01-07-03
BCG01-07-02
BCG01-18-06
BCG01-10-10
BCG01-08-01
BCG01-03-02

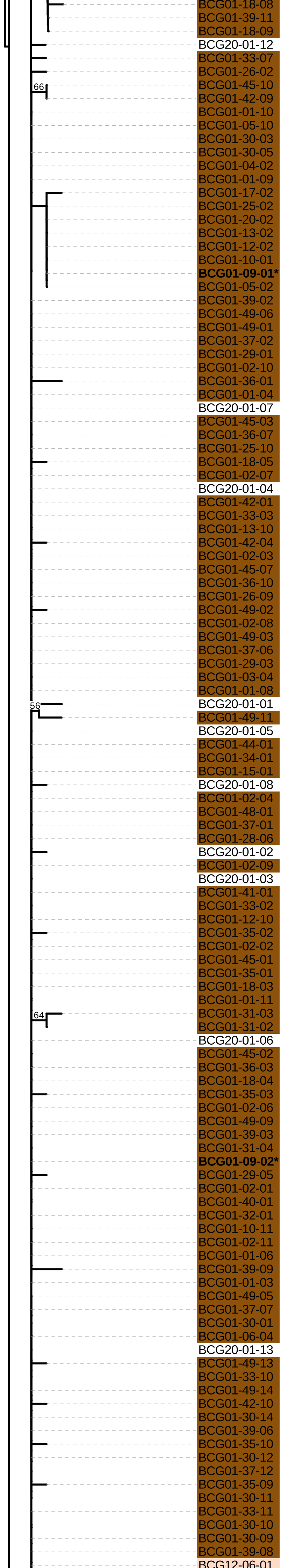

BCG01-18-08
BCG01-39-11
BCG01-18-09
BCG20-01-12
BCG01-33-07
BCG01-26-02
66
BCG01-45-10
BCG01-42-09
BCG01-01-10
BCG01-05-10
BCG01-30-03
BCG01-30-05
BCG01-04-02
BCG01-01-09
BCG01-17-02
BCG01-25-02
BCG01-20-02
BCG01-13-02
BCG01-12-02
BCG01-10-01
BCG01-09-01*
BCG01-05-02
BCG01-39-02
BCG01-49-06
BCG01-49-01
BCG01-37-02
BCG01-29-01
BCG01-02-10
BCG01-36-01
BCG01-01-04
BCG20-01-07
BCG01-45-03
BCG01-36-07
BCG01-25-10
BCG01-18-05
BCG01-02-07
BCG20-01-04
BCG01-42-01
BCG01-33-03
BCG01-13-10
BCG01-42-04
BCG01-02-03
BCG01-45-07
BCG01-36-10
BCG01-26-09
BCG01-49-02
BCG01-02-08
BCG01-49-03
BCG01-37-06
BCG01-29-03
BCG01-03-04
BCG01-01-08
56
BCG20-01-01
BCG01-49-11
BCG20-01-05
BCG01-44-01
BCG01-34-01
BCG01-15-01
BCG20-01-08
BCG01-02-04
BCG01-48-01
BCG01-37-01
BCG01-28-06
BCG20-01-02
BCG01-02-09
BCG20-01-03
BCG01-41-01
BCG01-33-02
BCG01-12-10
BCG01-35-02
BCG01-02-02
BCG01-45-01
BCG01-35-01
BCG01-18-03
BCG01-01-11
64
BCG01-31-03
BCG01-31-02
BCG20-01-06
BCG01-45-02
BCG01-36-03
BCG01-18-04
BCG01-35-03
BCG01-02-06
BCG01-49-09
BCG01-39-03
BCG01-31-04
BCG01-09-02*
BCG01-29-05
BCG01-02-01
BCG01-40-01
BCG01-32-01
BCG01-10-11
BCG01-02-11
BCG01-01-06
BCG01-39-09
BCG01-01-03
BCG01-49-05
BCG01-37-07
BCG01-30-01
BCG01-06-04
BCG20-01-13
BCG01-49-13
BCG01-33-10
BCG01-49-14
BCG01-42-10
BCG01-30-14
BCG01-39-06
BCG01-35-10
BCG01-30-12
BCG01-37-12
BCG01-35-09
BCG01-30-11
BCG01-33-11
BCG01-30-10
BCG01-30-09
BCG01-39-08
BCG12-06-01

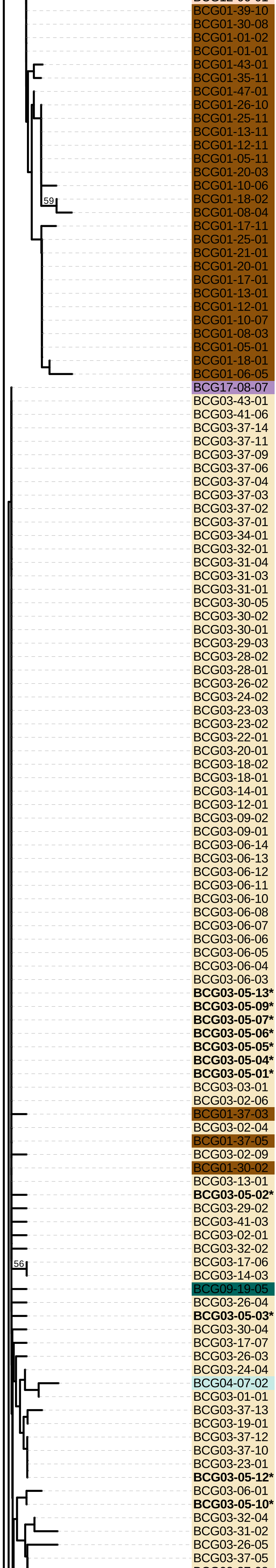
BCG01-39-10
BCG01-30-08
BCG01-01-02
BCG01-01-01
BCG01-43-01
BCG01-35-11
BCG01-47-01
BCG01-26-10
BCG01-25-11
BCG01-13-11
BCG01-12-11
BCG01-05-11
BCG01-20-03
BCG01-10-06
59
BCG01-18-02
BCG01-08-04
BCG01-17-11
BCG01-25-01
BCG01-21-01
BCG01-20-01
BCG01-17-01
BCG01-13-01
BCG01-12-01
BCG01-10-07
BCG01-08-03
BCG01-05-01
BCG01-18-01
BCG01-06-05
BCG17-08-07
BCG03-43-01
BCG03-41-06
BCG03-37-14
BCG03-37-11
BCG03-37-09
BCG03-37-06
BCG03-37-04
BCG03-37-03
BCG03-37-02
BCG03-37-01
BCG03-34-01
BCG03-32-01
BCG03-31-04
BCG03-31-03
BCG03-31-01
BCG03-30-05
BCG03-30-02
BCG03-30-01
BCG03-29-03
BCG03-28-02
BCG03-28-01
BCG03-26-02
BCG03-24-02
BCG03-23-03
BCG03-23-02
BCG03-22-01
BCG03-20-01
BCG03-18-02
BCG03-18-01
BCG03-14-01
BCG03-12-01
BCG03-09-02
BCG03-09-01
BCG03-06-14
BCG03-06-13
BCG03-06-12
BCG03-06-11
BCG03-06-10
BCG03-06-08
BCG03-06-07
BCG03-06-06
BCG03-06-05
BCG03-06-04
BCG03-06-03
BCG03-05-13*
BCG03-05-09*
BCG03-05-07*
BCG03-05-06*
BCG03-05-05*
BCG03-05-04*
BCG03-05-01*
BCG03-03-01
BCG03-02-06
BCG01-37-03
BCG03-02-04
BCG01-37-05
BCG03-02-09
BCG01-30-02
BCG03-13-01
BCG03-05-02*
BCG03-29-02
BCG03-41-03
BCG03-02-01
BCG03-32-02
56
BCG03-17-06
BCG03-14-03
BCG09-19-05
BCG03-26-04
BCG03-05-03*
BCG03-30-04
BCG03-17-07
BCG03-26-03
BCG03-24-04
BCG04-07-02
BCG03-01-01
BCG03-37-13
BCG03-19-01
BCG03-37-12
BCG03-37-10
BCG03-23-01
BCG03-05-12*
BCG03-06-01
BCG03-05-10*
BCG03-32-04
BCG03-31-02
BCG03-26-05
BCG03-37-05

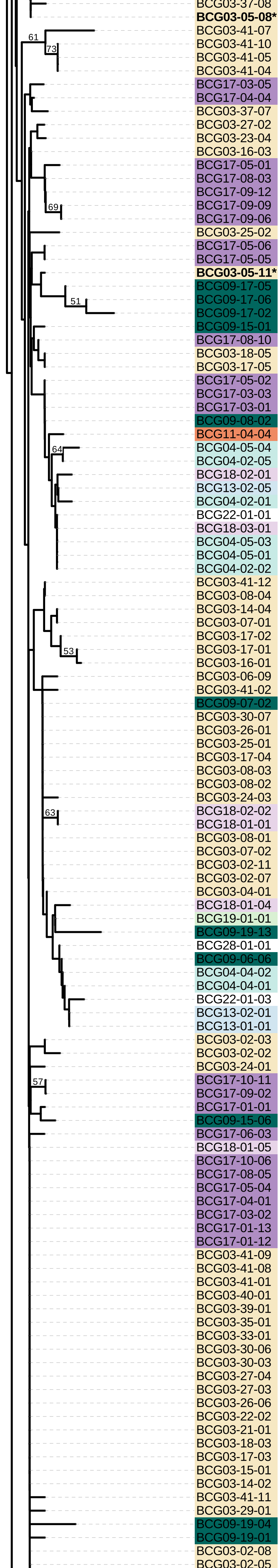

BCG03-37-08
BCG03-05-08*
61
BCG03-41-07
73
BCG03-41-10
BCG03-41-05
BCG03-41-04
BCG17-03-05
BCG17-04-04
BCG03-37-07
BCG03-27-02
BCG03-23-04
BCG03-16-03
BCG17-05-01
BCG17-08-03
BCG17-09-12
69
BCG17-09-09
BCG17-09-06
BCG03-25-02
BCG17-05-06
BCG17-05-05
BCG03-05-11*
BCG09-17-05
51
BCG09-17-06
BCG09-17-02
BCG09-15-01
BCG17-08-10
BCG03-18-05
BCG03-17-05
BCG17-05-02
BCG17-03-03
BCG17-03-01
BCG09-08-02
BCG11-04-04
64
BCG04-05-04
BCG04-02-05
BCG18-02-01
BCG13-02-05
BCG04-02-01
BCG22-01-01
BCG18-03-01
BCG04-05-03
BCG04-05-01
BCG04-02-02
BCG03-41-12
BCG03-08-04
BCG03-14-04
BCG03-07-01
BCG03-17-02
53
BCG03-17-01
BCG03-16-01
BCG03-06-09
BCG03-41-02
BCG09-07-02
BCG03-30-07
BCG03-26-01
BCG03-25-01
BCG03-17-04
BCG03-08-03
BCG03-08-02
BCG03-24-03
63
BCG18-02-02
BCG18-01-01
BCG03-08-01
BCG03-07-02
BCG03-02-11
BCG03-02-07
BCG03-04-01
BCG18-01-04
BCG19-01-01
BCG09-19-13
BCG28-01-01
BCG09-06-06
BCG04-04-02
BCG04-04-01
BCG22-01-03
BCG13-02-01
BCG13-01-01
BCG03-02-03
BCG03-02-02
BCG03-24-01
57
BCG17-10-11
BCG17-09-02
BCG17-01-01
BCG09-15-06
BCG17-06-03
BCG18-01-05
BCG17-10-06
BCG17-08-05
BCG17-05-04
BCG17-04-01
BCG17-03-02
BCG17-01-13
BCG17-01-12
BCG03-41-09
BCG03-41-08
BCG03-41-01
BCG03-40-01
BCG03-39-01
BCG03-35-01
BCG03-33-01
BCG03-30-06
BCG03-30-03
BCG03-27-04
BCG03-27-03
BCG03-26-06
BCG03-22-02
BCG03-21-01
BCG03-18-03
BCG03-17-03
BCG03-15-01
BCG03-14-02
BCG03-41-11
BCG03-29-01
BCG09-19-04
BCG09-19-01
BCG03-02-08
BCG03-02-05

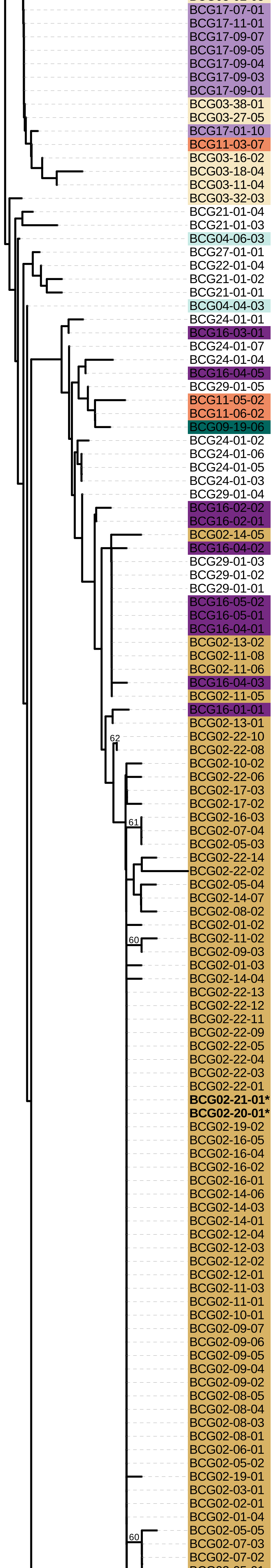
BCG17-07-01
BCG17-11-01
BCG17-09-07
BCG17-09-05
BCG17-09-04
BCG17-09-03
BCG17-09-01
BCG03-38-01
BCG03-27-05
BCG17-01-10
BCG11-03-07
BCG03-16-02
BCG03-18-04
BCG03-11-04
BCG03-32-03
BCG21-01-04
BCG21-01-03
BCG04-06-03
BCG27-01-01
BCG22-01-04
BCG21-01-02
BCG21-01-01
BCG04-04-03
BCG24-01-01
BCG16-03-01
BCG24-01-07
BCG24-01-04
BCG16-04-05
BCG29-01-05
BCG11-05-02
BCG11-06-02
BCG09-19-06
BCG24-01-02
BCG24-01-06
BCG24-01-05
BCG24-01-03
BCG29-01-04
BCG16-02-02
BCG16-02-01
BCG02-14-05
BCG16-04-02
BCG29-01-03
BCG29-01-02
BCG29-01-01
BCG16-05-02
BCG16-05-01
BCG16-04-01
BCG02-13-02
BCG02-11-08
BCG02-11-06
BCG16-04-03
BCG02-11-05
BCG16-01-01
BCG02-13-01
62
BCG02-22-10
BCG02-22-08
BCG02-10-02
BCG02-22-06
BCG02-17-03
BCG02-17-02
61
BCG02-16-03
BCG02-07-04
BCG02-05-03
BCG02-22-14
BCG02-22-02
BCG02-05-04
BCG02-14-07
BCG02-08-02
BCG02-01-02
60
BCG02-11-02
BCG02-09-03
BCG02-01-03
BCG02-14-04
BCG02-22-13
BCG02-22-12
BCG02-22-11
BCG02-22-09
BCG02-22-05
BCG02-22-04
BCG02-22-03
BCG02-22-01
BCG02-21-01*
BCG02-20-01*
BCG02-19-02
BCG02-16-05
BCG02-16-04
BCG02-16-02
BCG02-16-01
BCG02-14-06
BCG02-14-03
BCG02-14-01
BCG02-12-04
BCG02-12-03
BCG02-12-02
BCG02-12-01
BCG02-11-03
BCG02-11-01
BCG02-10-01
BCG02-09-07
BCG02-09-06
BCG02-09-05
BCG02-09-04
BCG02-09-02
BCG02-08-05
BCG02-08-04
BCG02-08-03
BCG02-08-01
BCG02-06-01
BCG02-05-02
BCG02-19-01
BCG02-03-01
BCG02-02-01
BCG02-01-04
BCG02-05-05
60
BCG02-07-03
BCG02-07-02

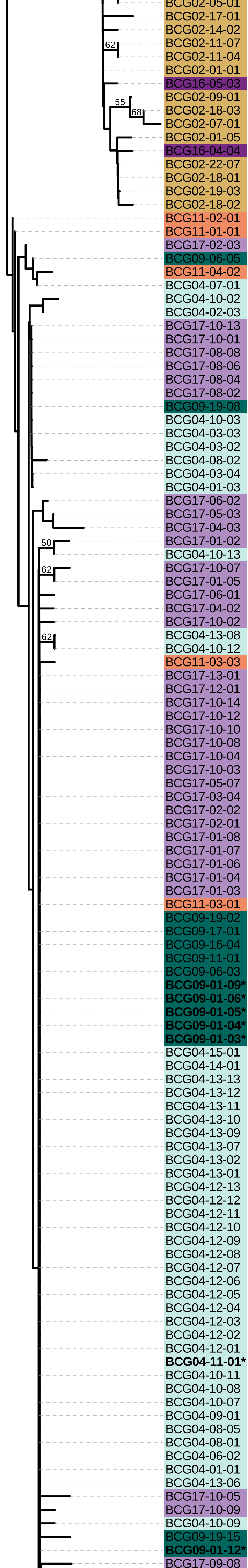

BCG02-05-01
BCG02-17-01
BCG02-14-02
62
BCG02-11-07
BCG02-11-04
BCG02-01-01
BCG16-05-03
55
BCG02-09-01
68
BCG02-18-03
BCG02-07-01
BCG02-01-05
BCG16-04-04
BCG02-22-07
BCG02-18-01
BCG02-19-03
BCG02-18-02
BCG11-02-01
BCG11-01-01
BCG17-02-03
BCG09-06-05
BCG11-04-02
BCG04-07-01
BCG04-10-02
BCG04-02-03
BCG17-10-13
BCG17-10-01
BCG17-08-08
BCG17-08-06
BCG17-08-04
BCG17-08-02
BCG09-19-08
BCG04-10-03
BCG04-03-03
BCG04-03-02
BCG04-08-02
BCG04-03-04
BCG04-01-03
BCG17-06-02
BCG17-05-03
BCG17-04-03
BCG17-01-02
50
BCG04-10-13
62
BCG17-10-07
BCG17-01-05
BCG17-06-01
BCG17-04-02
BCG17-10-02
62
BCG04-13-08
BCG04-10-12
BCG11-03-03
BCG17-13-01
BCG17-12-01
BCG17-10-14
BCG17-10-12
BCG17-10-10
BCG17-10-08
BCG17-10-04
BCG17-10-03
BCG17-05-07
BCG17-03-04
BCG17-02-02
BCG17-02-01
BCG17-01-08
BCG17-01-07
BCG17-01-06
BCG17-01-04
BCG17-01-03
BCG11-03-01
BCG09-19-02
BCG09-17-01
BCG09-16-04
BCG09-11-01
BCG09-06-03
BCG09-01-09*
BCG09-01-06*
BCG09-01-05*
BCG09-01-04*
BCG09-01-03*
BCG04-15-01
BCG04-14-01
BCG04-13-13
BCG04-13-12
BCG04-13-11
BCG04-13-10
BCG04-13-09
BCG04-13-07
BCG04-13-02
BCG04-13-01
BCG04-12-13
BCG04-12-12
BCG04-12-11
BCG04-12-10
BCG04-12-09
BCG04-12-08
BCG04-12-07
BCG04-12-06
BCG04-12-05
BCG04-12-04
BCG04-12-03
BCG04-12-02
BCG04-12-01
BCG04-11-01*
BCG04-10-11
BCG04-10-08
BCG04-10-07
BCG04-09-01
BCG04-08-05
BCG04-08-01
BCG04-06-02
BCG04-01-01
BCG04-13-06
BCG17-10-05
BCG17-10-09
BCG04-10-09
BCG09-19-15
BCG09-01-12*
BCG17-09-08

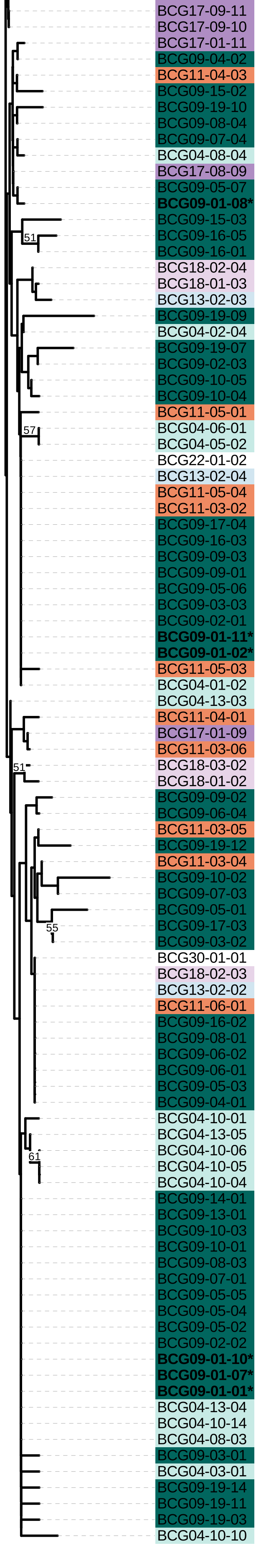

BCG17-09-11
BCG17-09-10
BCG17-01-11
BCG09-04-02
BCG11-04-03
BCG09-15-02
BCG09-19-10
BCG09-08-04
BCG09-07-04
BCG04-08-04
BCG17-08-09
BCG09-05-07
BCG09-01-08*
BCG09-15-03
51
BCG09-16-05
BCG09-16-01
BCG18-02-04
BCG18-01-03
BCG13-02-03
BCG09-19-09
BCG04-02-04
BCG09-19-07
BCG09-02-03
BCG09-10-05
BCG09-10-04
BCG11-05-01
57
BCG04-06-01
BCG04-05-02
BCG22-01-02
BCG13-02-04
BCG11-05-04
BCG11-03-02
BCG09-17-04
BCG09-16-03
BCG09-09-03
BCG09-09-01
BCG09-05-06
BCG09-03-03
BCG09-02-01
BCG09-01-11*
BCG09-01-02*
BCG11-05-03
BCG04-01-02
BCG04-13-03
BCG11-04-01
BCG17-01-09
BCG11-03-06
51
BCG18-03-02
BCG18-01-02
BCG09-09-02
BCG09-06-04
BCG11-03-05
BCG09-19-12
BCG11-03-04
BCG09-10-02
BCG09-07-03
BCG09-05-01
55
BCG09-17-03
BCG09-03-02
BCG30-01-01
BCG18-02-03
BCG13-02-02
BCG11-06-01
BCG09-16-02
BCG09-08-01
BCG09-06-02
BCG09-06-01
BCG09-05-03
BCG09-04-01
BCG04-10-01
BCG04-13-05
BCG04-10-06
61
BCG04-10-05
BCG04-10-04
BCG09-14-01
BCG09-13-01
BCG09-10-03
BCG09-10-01
BCG09-08-03
BCG09-07-01
BCG09-05-05
BCG09-05-04
BCG09-05-02
BCG09-02-02
BCG09-01-10*
BCG09-01-07*
BCG09-01-01*
BCG04-13-04
BCG04-10-14
BCG04-08-03
BCG09-03-01
BCG04-03-01
BCG09-19-14
BCG09-19-11
BCG09-19-03
BCG04-10-10

Figure S2 | Phylogenetic tree inferred from 1007 copies of the 16S rRNA gene of 202 bacteria of the *B. cereus* group. A total of 1007 copies of the 16S rRNA gene were complete from site 352 to site 1051 in the complete 16S rRNA gene, and used to construct the tree. *B. subtilis* ATCC 6051^T (BSU6051_0082022) was used as the outgroup. The 16S rRNA genes extracted from type strains are marked by the asterisk symbol (*) and printed in bold font. See the main text for more descriptions.

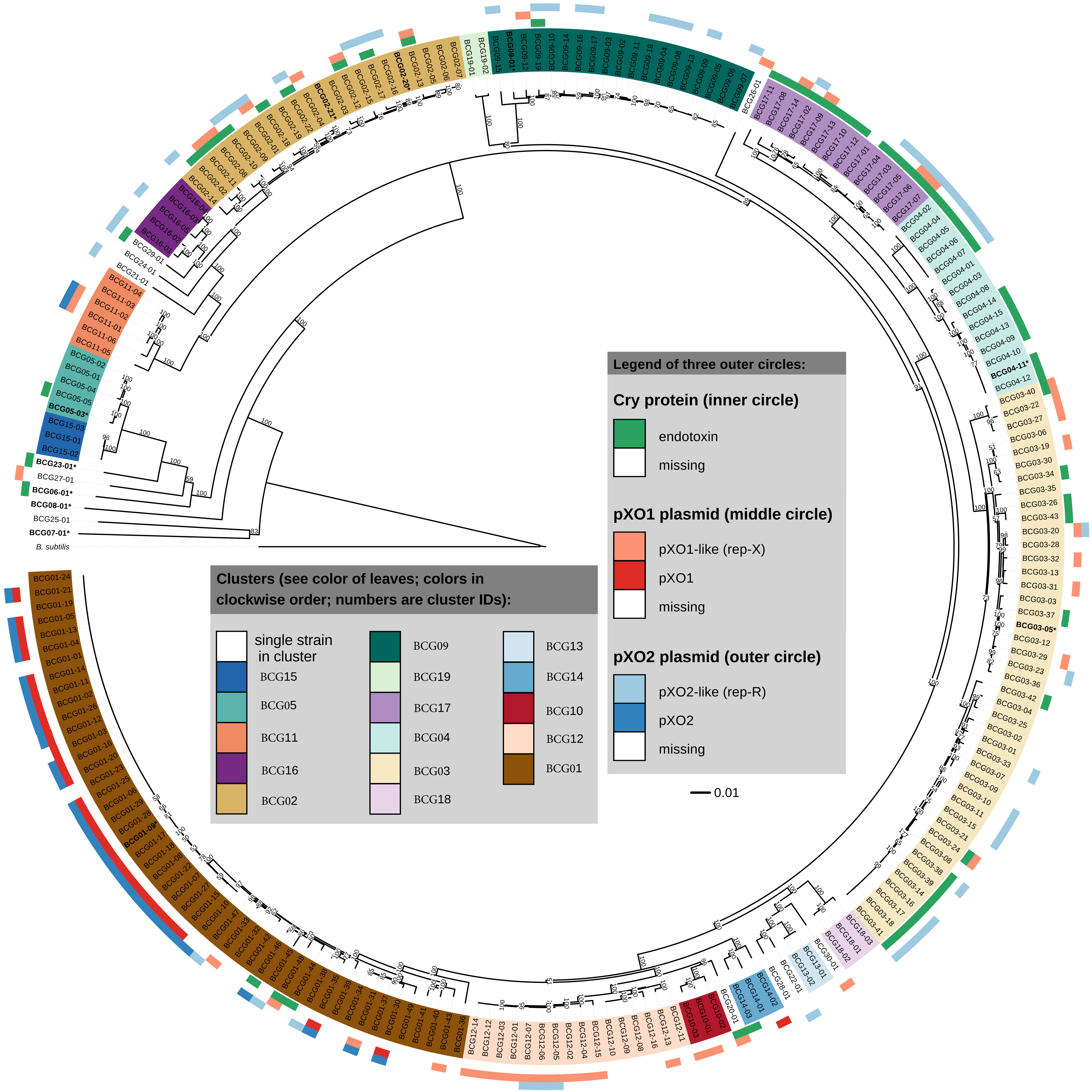

Legend of three outer circles:
Cry protein (inner circle)
endotoxin
missing
pXO1 plasmid (middle circle)
pXO1-like (rep-X)
pXO1
missing
pXO2 plasmid (outer circle)
pXO2-like (rep-R)
pXO2
missing
0.01
Clusters (see color of leaves; colors in clockwise order; numbers are cluster IDs):
single strain in cluster
BCG15
BCG05
BCG11
BCG16
BCG02
BCG09
BCG19
BCG17
BCG04
BCG03
BCG18
BCG13
BCG14
BCG10
BCG12
BCG01
B. subtilis

Figure S3 | nMLSA-based phylogenetic tree of 224 bacteria of the *B. cereus* group using 20 housekeeping genes. Numbers on branches are bootstrap support values from 1,000 replicates given in percent. Branches are scaled in terms of the expected number of substitutions per site. Leaves are colored according to their affiliation to clusters (compare Figure 1). The three outer circles show whether or not the (i) *cry* locus, (ii) pXO1(-like) plasmid and/or the (iii) pXO2(-like) plasmid are found. Type strains are printed in bold font and marked by an asterisk (*). *Bacillus subtilis* ATCC 6051^T (CP003329) was used as the outgroup.

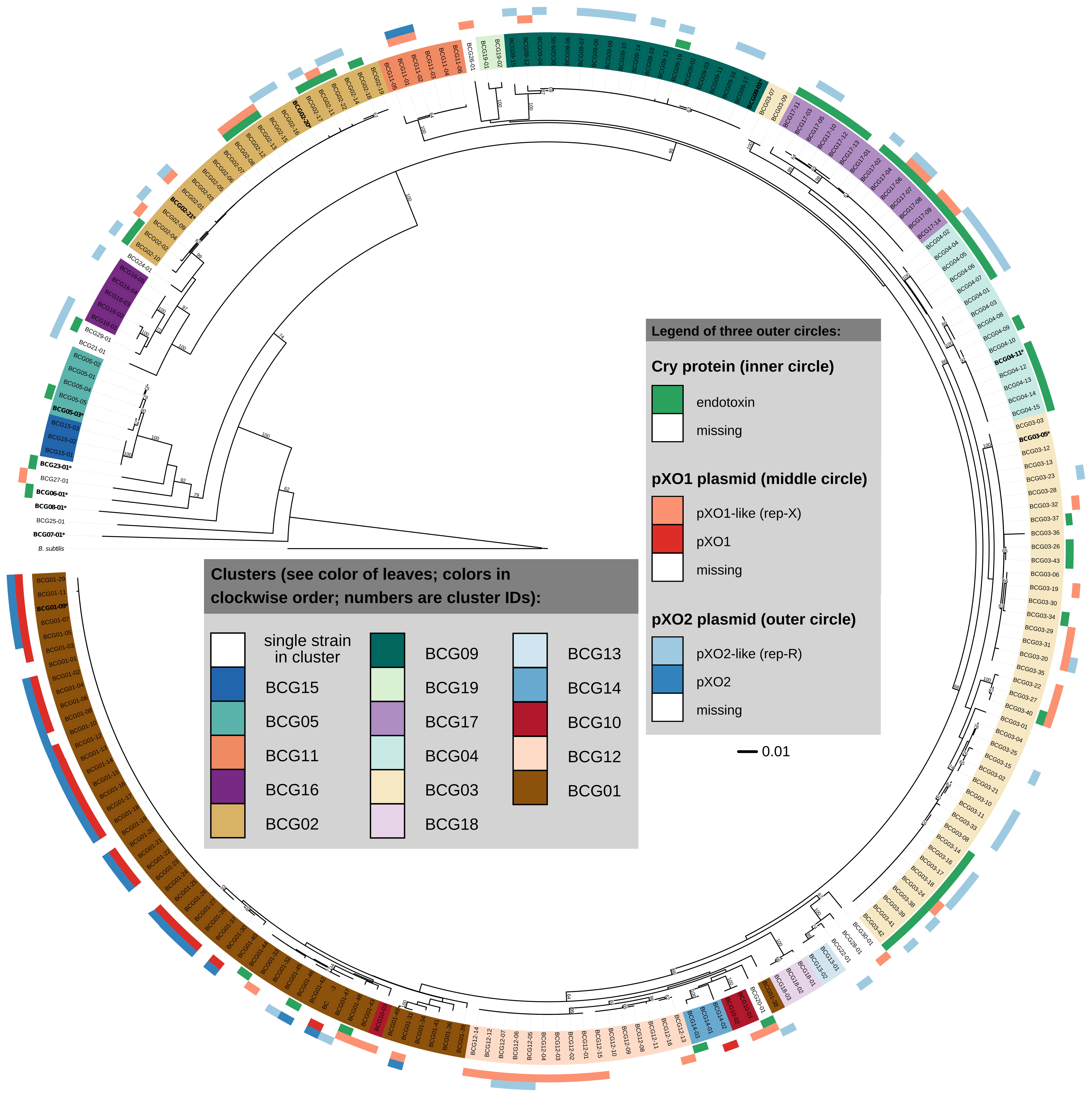
Legend of three outer circles:
Cry protein (inner circle)
endotoxin
missing
pXO1 plasmid (middle circle)
pXO1-like (rep-X)
pXO1
missing
pXO2 plasmid (outer circle)
pXO2-like (rep-R)
pXO2
missing
0.01
Clusters (see color of leaves; colors in clockwise order; numbers are cluster IDs):
single strain in cluster
BCG15
BCG05
BCG11
BCG16
BCG02
BCG09
BCG19
BCG17
BCG04
BCG03
BCG18
BCG13
BCG14
BCG10
BCG12
BCG01
B. subtilis

Figure S4 | Phylogenetic trees inferred from the *ccpA* gene of 224 bacteria of the *B. cereus* group. Numbers on branches are bootstrap support values from 1,000 replicates given in percent. Branches are scaled in terms of the expected number of substitutions per site. Leaves are colored according to their affiliation to clusters (compare Figure 1). The three outer circles show whether or not the (i) *cry* locus, (ii) pXO1(-like) plasmid and/or the (iii) pXO2(-like) plasmid are found. Type strains are printed in bold font and marked by an asterisk (*). *Bacillus subtilis* ATCC 6051^{T} (CP003329) was used as the outgroup.
